# Opposing Effects of cAMP and T259 Phosphorylation on Plasma Membrane Diffusion of the Water Channel Aquaporin-5 in Madin-Darby Canine Kidney Cells

**DOI:** 10.1371/journal.pone.0133324

**Published:** 2015-07-28

**Authors:** Jennifer S. Koffman, Eva C. Arnspang, Saw Marlar, Lene N. Nejsum

**Affiliations:** Department of Molecular Biology and Genetics and Interdisciplinary Nanoscience Center, Aarhus University, Aarhus, Denmark; University of Bari Aldo Moro, ITALY

## Abstract

Aquaporin-5 (AQP5) facilitates passive water transport in glandular epithelia in response to secretory stimuli via intracellular pathways involving calcium release, cAMP and protein kinase A (PKA). In epithelial plasma membranes, AQP5 may be acutely regulated to facilitate water transport in response to physiological stimuli by changes in protein modifications, interactions with proteins and lipids, nanoscale membrane domain organization, and turnover rates. Such regulatory mechanisms could potentially be associated with alteration of diffusion behavior, possibly resulting in a change in the plasma membrane diffusion coefficient of AQP5. We aimed to test the short-term regulatory effects of the above pathways, by measuring lateral diffusion of AQP5 and an AQP5 phospho-mutant, T259A, using k-space Image Correlation Spectroscopy of quantum dot- and EGFP-labeled AQP5. Elevated cAMP and PKA inhibition significantly decreased lateral diffusion of AQP5, whereas T259A mutation showed opposing effects; slowing diffusion without stimulation and increasing diffusion to basal levels after cAMP elevation. Thus, lateral diffusion of AQP5 is significantly regulated by cAMP, PKA, and T259 phosphorylation, which could be important for regulating water flow in glandular secretions.

## Introduction

Epithelia are functional barriers lining exterior surfaces, tubes, and glands, allowing selective transport of water, ions, and other solutes [1].

Aquaporin water channels (AQP) are homotetrameric transmembrane proteins which facilitate trans-epithelial water transport across plasma membranes in response to osmotic gradients. 13 mammalian homologs (AQP0-12) have been identified and are expressed in a wide range of tissues including eye, kidney, secretory glands, airways and lungs, and brain (for review [2]).

AQP5 is the main water channel in secretory glands [1, 3–10]. AQP5 has been localized to secretory apical [1, 4, 8–10] and intercellular canalicular membranes [1, 4, 8] and sometimes basolateral membranes [3], in the airways and lungs [4, 9] as well as sweat [3, 10] and salivary glands [1, 4, 7, 8] of mouse [3, 9, 10], rat [1, 4, 7], and humans [3, 8], where it is involved in osmotic-driven water transport across glandular epithelial membranes [3, 6, 9, 11]. Glandular secretion is mediated by stimulation of muscarinic and adrenergic receptors as well as neuropeptide release, leading either to generation of inositol 1,4,5-triphosphate and diacylglycerol followed by transient increase of calcium in the cytoplasm or cAMP and PKA dependent signaling (for reviews [12, 13]).

AQP5 expression levels and subcellular localization are regulated by increase in cAMP in a dose- and time-dependent manner [14]; short-term incubation with cAMP caused AQP5 surface internalization and long-term treatment increased the AQP5 plasma membrane abundance [15]. Effects of cAMP on AQP5 mRNA and protein could both be prevented by the PKA inhibitor H89 [14, 15].

AQP5 is phosphorylated upon increased cAMP [15]. Short-term cAMP stimulation mediated phosphorylation of flag-tagged human AQP5 but not a S156A mutant [16], whereas another study found that PKA phosphorylated AQP5 upon cAMP stimulation on T259 and not on S156 [17]. Moreover, T259 phosphorylation was transiently increased in mouse submandibular and parotid glands after stimulation of saliva secretion with β-adrenergic agonist isoproterenol (but not pilocarpine) [17]. Phosphorylation did not seem to change AQP5 water permeability [15], thus, phosphorylation may serve a regulatory function in respect to expression levels and plasma membrane life-time, as is seen for the homologous AQP2, where S256 and S269 phosphorylation has been shown to regulate plasma membrane turn-over [18, 19].

Plasma membrane proteins may be dynamically regulated in the plasma membrane by posttranslational modifications, protein and lipid interactions, and organization into functional microdomains that are not easily detected, but could affect the diffusion behavior of the protein (for review [20]) and potentially be measured as a change in the diffusion coefficient. The diffusion coefficient of rat AQP2-EGFP, but not AQP1-EGFP, measured by fluorescence recovery after photobleaching (FRAP) over 10 minutes decreased 10-fold from 0.009 μm^2^/s in the plasma membrane of cell-cell contacts of LLC-PK1 kidney cells after short-term stimulation with the cAMP elevating agent forskolin [21], showing that changes in AQP2 diffusion are associated with elevated cAMP, known to regulate apical plasma membrane association of AQP2 in kidney collecting duct cells [22].We speculated whether AQP5 is dynamically regulated in glandular epithelial membranes to facilitate secretion, which could be associated with changes in the AQP5 diffusion coefficient. Thus, the aim of this work was to measure average diffusion coefficients of AQP5-EGFP in the epithelial cell line MDCK [23–25] upon relevant physiological stimulations. We accomplished this by employing k-space Image Correlation Spectroscopy (kICS) of image sequences from time-lapse microscopy of quantum dot (QD)- and EGFP-labeled AQP5. kICS is a recently developed correlation technique which computes correlations between image frames in an image series; and from this determines particle motion dynamics. In kICS, each individual image is first 2D Fourier transformed to its k-space spatial frequency representation, and the time correlations are then computed in the image stack in k-space. The k-space time correlation decay is fit to an analytical model and from this the diffusion coefficient or flow speed is extracted [26].

## Materials and Methods

### DNA constructs and site-directed ligase-independent mutagenesis

AQP5-EGFP-N2 was a gift from A. Aperia, Karolinska Institutet, Sweden [27]. A myc-tag flanked by alanine residues (A-EQKLISEED-A) was inserted into the second extracellular loop of AQP5 after amino acid N117 (AQP5-myc-EGFP) and the T259A mutation was introduced (AQP5-myc-T259A-EGFP) using site-directed ligase-independent mutagenesis [28]. The myc insertion did not change cellular localization of AQP5. Primers are listed below.

AQP5-myc-EGFP:

F_short_: CTGGCCGTCAACGCGCTC

F_tail_: GCGGAACAAAAACTTATTTCTGAAGAAGATCTGGCGCTGGCCGTCAACGCGCTC

R_short_: ATTGCCCCGGGCATTGAG

R_tail_: CGCCAGATCTTCTTCAGAAATAAGTTTTTGTTCCGCATTGCCCCGGGCATTGAG

AQP5-myc-T259A-EGFP:

F_short_: CTGACCACCCGCGAATTC

F_tail_: CGGAAGAAGGCCATGGAGCTGACCACCCGCGAATTC

R_short_: CTCTTCCCGCTGCTCCTC

R_tail_: CTCCATGGCCTTCTTCCGCTCTTCCCGCTGCTCCTC

### Cell culture

MDCK GII cells [29, 30] and MDCK GII stably expressing AQP5-EGFP [25] and AQP5-myc-EGFP were cultured in Dulbecco’s Modified Eagle Medium (DMEM) low (1 g/L glucose) (Gibco, Thermo Fischer Scientific, Waltham, MA) supplemented with 10% FBS (Gibco) and 1X PSK (0.5 U/ml Penicillin G sodium salt (Sigma-Aldrich, St. Louis, MO), 0.5 mg/ml streptomycin sulfate (Gibco), and 1 mg/ml kanamycin sulfate (Gibco)). Cells were kept at 37°C under 5% CO_2_. Stable MDCK AQP5-myc-EGFP cells were generated by selection in 500 μg/ml G418 geneticin sulfate (Gibco). Live cell imaging was performed in phenol-red free DMEM low (Gibco) supplemented with 10% FBS, 1X PSK, and 25 mM HEPES (Gibco) (see below).

### Preparation of cells for QD-labeling and time-lapse microscopy

For live cell imaging, cells were seeded at subconfluency onto rat-tail collagen-coated coverslips the day before imaging. For comparison of AQP5-myc-EGFP wild-type (wt) and AQP5-myc-T259A-EGFP, cells were transiently transfected two days before imaging and split onto collagen-coated coverslips the day before imaging. QD-labeling was performed in culture media [31].

### Drug treatments

Cells were treated with 50 μM forskolin (Sigma-Aldrich) or 30 μM H89 dihydrochloride hydrate in DMSO (Sigma-Aldrich) for 30 min before QD-labeling and imaging. 10 μM calcium ionophore A23187 in DMSO (Sigma-Aldrich) was added to pre-labeled cells for 5 min before imaging and 5 mM methyl-β-cyclodextrin (MBCD) in water was added to cells for 15 min before labeling and imaging.

### Time-lapse microscopy

Imaging of QD-labeled AQP5-myc-EGFP was performed on a Zeiss Axiovert 200M inverted fluorescence microscope equipped with a Xenon lamp and a 37°C heated stage using a 63x/1.45 NA oil objective (Carl Zeiss, Jena, Germany). A single EGFP image followed by a QD image sequence of 500 frames with 20 ms integration time at a frame rate of 11.91 Hz were acquired with a Photometrics CoolSnap HQ cooled CCD (Roper Scientific, Martinsried, Germany) controlled by MetaMorph (Molecular Devices, Sunnyvale, CA). For EGFP a D480/40 excitation filter and a D535/50 emission filter was used, whereas for QDs, a D560/40 excitation filter and D630/60 emissions filter (Chroma Technology, Bellows Falls, VT) was used.

Image sequences of EGFP were acquired on an Olympus IX-81 inverted fluorescence microscope equipped with a 100 W Hg arc lamp at room temperature (media was 30°C during acquisition) using a 150x/1.45 NA oil TIRF objective (Olympus, Tokyo, Japan). Image series of 600 frames with 20 ms integration were acquired at a frame rate of 19.89 Hz with an Andor DV887-ECS EMCCD camera (Andor, Belfast, Northern Ireland). For detection of EGFP, a combination of a 470/40 nm excitation bandpass filter, a Q495LP dichroic filter, and a HQ510LP emission filter (Chroma Technology) was used.

### Measurement of diffusion coefficients by kICS analysis

Time-lapse image sequences were cropped without any post-acquisition processing using ImageJ [32]. Crops were analyzed in MATLAB (The MathWorks, Natick, MA) with the kICS code [26]. Calculation of the diffusion coefficient from QD image sequences was made with maximum number of time lags (τ) set to 12 and the maximum k^2^ value was 30. Analysis of the diffusion coefficient from EGFP image sequences was performed with maximum τ set to 6 and the maximum k^2^ value was 20. Results are shown as the average diffusion coefficient D in μm^2^/s and an average diffusion plot of the time decay-Dt in μm^2^ versus time t in seconds (s) over all crops for each condition. In the diffusion plot, the slope of the line is-D. The minus sign is from the slope of the line fit in the k2 plots. The value, where the trend line in the diffusion plot crosses the y-axis is according to the formula: $-(D\tau +\frac{\omega^{2}}{4})$ where τ is the timelag and ω is the width of the point spread function. So even for τ = 0, the expression has a value as ω is always larger than 0. All formulas are described in [26]. Thus parallel lines have the same diffusion coefficients, whereas nonparallel lines have different diffusion coefficients. For each condition, a minimum of 6 crops from minimum 5 different cells for transiently transfected cells and a minimum of 9 crops from minimum 7 different cells for stable cell lines were pooled from at least three experiments.

### Statistics

Values are presented as means ± standard deviations. Comparisons of diffusion coefficients were evaluated using unpaired *t*-test. *p*-values < 0.05 were considered significant.

## Results and Discussion

### Elevation of cAMP and PKA inhibition both reduce the AQP5 diffusion coefficient

Besides long-term regulation of AQP5 plasma membrane abundance to facilitate water transport associated with glandular secretion, AQP5 could be regulated on a fast time-scale within epithelial plasma membranes by post-translational modifications, interactions with other proteins and lipids, and incorporation into microdomains which could modulate function and/or rate of accumulation and endocytosis/turn-over. Such events could be associated with a dynamic change in AQP5 diffusion behavior within the plasma membrane.

This study aimed to determine the plasma membrane diffusion coefficient of AQP5 under normal resting conditions and after physiological relevant stimulations. MDCK cells have previously been used for diffusion measurements of AQPs [21, 33–35] and were chosen as a general epithelial cell model system that does not express endogenous AQP5. QD-labeling, previously employed to investigate plasma membrane diffusion of AQP1 and AQP4 by single particle tracking analysis [33, 34] and AQP3 by kICS [31], was compared to simply using the EGFP-label, for extracting average diffusion coefficients of AQP5 by kICS analysis of epifluorescence time-lapse series.

**Fig 1:**
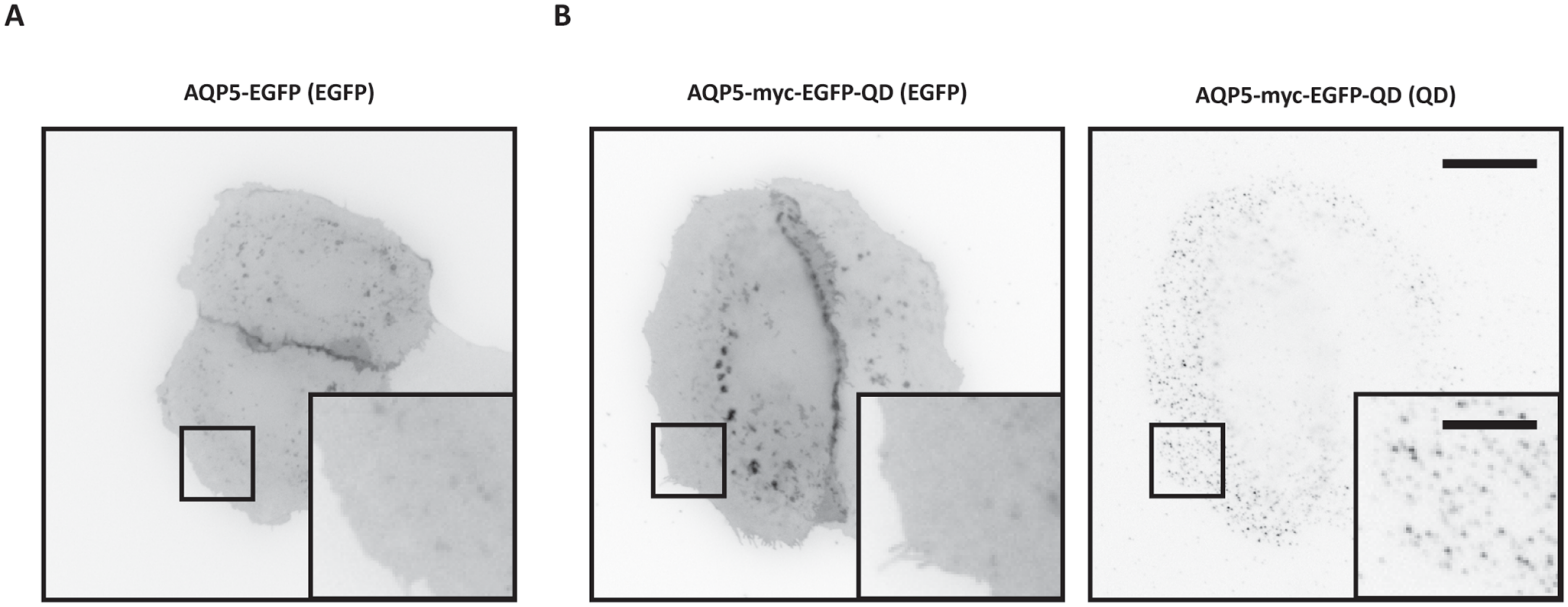
AQP5 expression in MDCK cells. (A-B) Epifluorescent images show the subcellular localization of AQP5 in live MDCK cells. Fluorescence is shown in inverted contrast. (A) Untreated MDCK AQP5-EGFP cells. (B) Untreated MDCK AQP5-myc-EGFP cells labeled with QDs. Insets (boxed areas) highlight the homogenous plasma membrane distribution of AQP5 in the flat portion of the cell. To compare cellular localization, AQP5-EGFP was acquired with 100 ms integration, whereas AQP5-myc-EGFP was acquired with 20 ms integration on a Zeiss Axiovert 200M inverted epifluorescence microscope. EGFP images were adjusted to the same minimum and maximum displayed intensity values. Scale bars are 20 μm and 7 μm (*insets*).

In subconfluent MDCK AQP5-EGFP cells, AQP5-EGFP was distributed homogenously throughout the entire plasma membrane (Fig 1A) and the same localization pattern was also observed in MDCK cells stably expressing AQP5-myc-EGFP (Fig 1B). QDs were evenly distributed on the free surface of the cells (Fig 1B). kICS analysis was performed on image crops that included only the flat part of the cell where lateral diffusion can be assumed, avoiding membrane overlaying the cell body and nucleus where QDs were out-of focus. Aggregation or internalization of QDs was not apparent within the set time limit for microscopy analysis after QD-labeling was completed, but could be observed over extended time periods (not shown).

**Fig 2:**
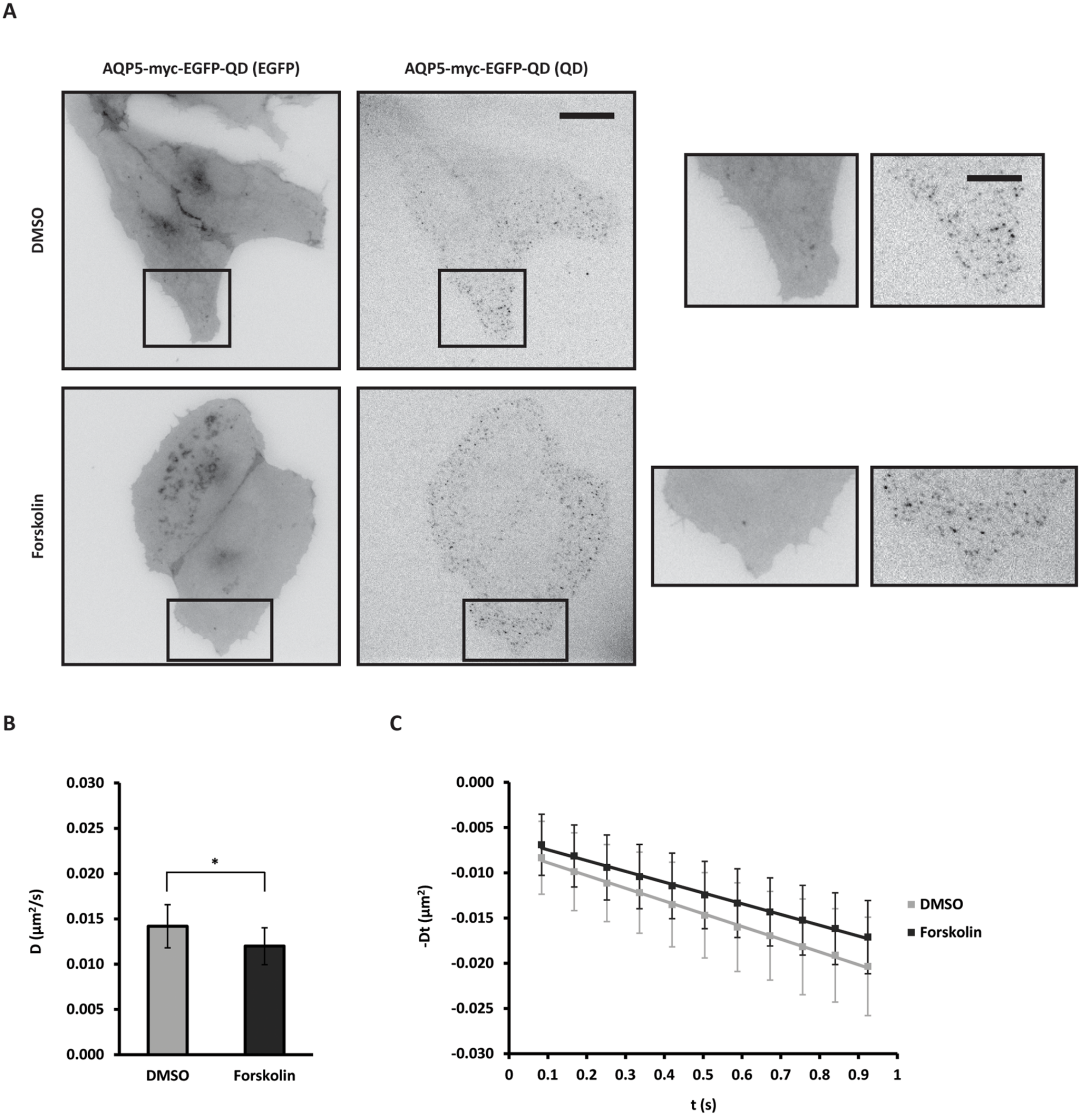
Elevation of cAMP reduces the AQP5 diffusion coefficient. (A-C) MDCK cells stably expressing AQP5-myc-EGFP were treated with 50 μM forskolin or vehicle (DMSO) for 30 min before labeling with QDs and followed by time-lapse microscopy with 20 ms integration for 500 frames at 11.91 Hz. Crops of image sequences were subjected to kICS analysis to determine average diffusion coefficients. (A) Example of an AQP5-myc-EGFP image and frame from the QD time-lapse from DMSO and forskolin treated cells, including crops (boxed areas) used for kICS analysis. Fluorescence is shown inverted contrast. EGFP images and QD frames were adjusted to the same minimum and maximum displayed intensity values for the two conditions. Scale bars are 20 μm and 10 μm (*crops*). (B) Graph showing the average diffusion coefficient, D, in μm^2^/s over all crops for cells treated with forskolin and DMSO. (C) Diffusion plots showing time decay, -Dt, in μm^2^ versus time, t, in s averaged over all crops for forskolin and DMSO treated cells. Values represent the mean ± standard deviation. * indicates *p* < 0.05.

Forskolin treatment to elevate cAMP significantly reduced the diffusion coefficient of QD-labeled AQP5 to 84.5% of the control (0.0142 ± 0.0024 μm^2^/s vs. 0.0120 ± 0.0020 μm^2^/s for DMSO and forskolin, respectively, *p* < 0.05) (Fig 2B and 2C and Table 1), example of an EGFP image and frame from a QD time-lapse used to extract average diffusion coefficients is shown in Fig 2A from a forskolin and DMSO treated cell, including a crop used for kICS analysis. Forskolin had no apparent effect on AQP5 subcellular localization and extent of QD-labeling as compared to DMSO control cells and untreated cells (Figs 2A and 1B). DMSO did not influence the average diffusion coefficient of QD-labeled AQP5 (0.0131 ± 0.0031 μm^2^/s vs. 0.0129 ± 0.0022 μm^2^/s for untreated and DMSO, respectively) (S1 Fig and Table 1), nor did the inserted myc-tag (0.0332 ± 0.0110 μm^2^/s vs. 0.0295 ± 0.0089 μm^2^/s, for AQP5-EGFP and AQP5-myc-EGFP, respectively) (S2 Fig and Table 1). In MDCK AQP5-myc-EGFP cells without QD-labeling the AQP5 diffusion coefficient was reduced to 79.1% of the control in response to forskolin (0.0282 ± 0.0109 μm^2^/s vs. 0.0223 ± 0.0094 μm^2^/s for DMSO and forskolin, respectively, *p* <0.05) (S2 Fig and Table 1).

**Table 1:** Summary of AQP5 diffusion coefficients.

AQP5 label	Cells	Condition	D (um^2^/s)	% of control	*p*-value
**QD**	AQP5-myc-EGFP	Untreated	0.0131 ± 0.0031		
	AQP5-myc-EGFP	DMSO	0.0129 ± 0.0022	98.3% (DMSO vs. Untreated)	0.8121
	AQP5-myc-EGFP	DMSO	0.0142 ± 0.0024		
	AQP5-myc-EGFP	Forskolin	0.0120 ± 0.0020	84.5% (Forskolin vs. DMSO)	0.0127*
	AQP5-myc-EGFP	DMSO	0.0160 ± 0.0042		
	AQP5-myc-EGFP	H89	0.0134 ± 0.0033	83.5% (H89 vs. DMSO)	0.0407*
	AQP5-myc-EGFP	DMSO	0.0103 ± 0.0026		
	AQP5-myc-EGFP	A23187	0.0101 ± 0.0022	98.5% (A23187 vs. DMSO)	0.8512
	AQP5-myc-EGFP	Water	0.0132 ± 0.0035		
	AQP5-myc-EGFP	MBCD	0.0125 ± 0.0039	94.8% (MBCD vs. Water)	0.6626
	AQP5-myc-EGFP (wt)	DMSO	0.0164 ± 0.0038	70.1% (wt: Forskolin vs. DMSO)	0.0137*
	AQP5-myc-EGFP (wt)	Forskolin	0.0115 ± 0.0031	141.7% (Forskolin: T259A vs. wt)	0.0136*
	AQP5-myc-T259A-EGFP	DMSO	0.0117 ± 0.0025	71.3% (DMSO: T259A vs. wt)	0.0044*
	AQP5-myc-T259A-EGFP	Forskolin	0.0163 ± 0.0041	139.3% (T259A: Forskolin vs. DMSO)	0.0046*
**EGFP**	AQP5-EGFP	Untreated	0.0332 ± 0.0110	88.9% (Untreated: AQP5-myc-EGFP vs. AQP5-EGFP)	0.3233
	AQP5-myc-EGFP	Untreated	0.0295 ± 0.0089	75.7% (AQP5-myc-EGFP: Forskolin vs. Untreated)	0.0227*
	AQP5-myc-EGFP	DMSO	0.0282 ± 0.0109	95.6% (AQP5-myc-EGFP: DMSO vs. Untreated)	0.6999
	AQP5-myc-EGFP	Forskolin	0.0223 ± 0.0094	79.1% (AQP5-myc-EGFP: Forskolin vs. DMSO)	0.0399*

* Indicates *p*-values < 0.05, which are considered significant.

The diffusion coefficients of AQP5-myc-EGFP without QD-labeling were larger, suggesting that addition of the QD adds bulk to the protein which slows the diffusion as seen in other studies [31]. Nevertheless, the same relative change upon treatment with forskolin was observed. The diffusion coefficients of QD-labeled AQP5 were in the same range as QD-labeled AQP3 as well as those measured by EGFP imaging were similar for AQP5 and AQP3 [31, 36, 37]. Average diffusion coefficients determined from QD-labeling had smaller standard deviations compared to EGFP-labeling. This could be due to only a subset of AQP5 proteins specifically in the plasma membrane being labeled by QDs, whereas the entire pool of AQP5 proteins in the cell were labeled with EGFP. In addition, QDs are brighter than EGFP, which increases signal vs. background intensities. The diffusion coefficients of QD-labeled AQP4 and AQP1 in COS-7 cells were slightly larger [33, 34]. This may be explained by the additional EGFP-tag present in both AQP3 and AQP5 or could represent cell type related differences. Diffusion coefficients of AQP4 and AQP1 were both considerably higher in COS-7 cells compared to MDCKI and MDCKII cells, which was proposed to be a membrane crowding effect due to different lipid composition and presence of microvilli on MDCK cells [33, 34]. In comparison, the diffusion coefficient of AQP3 measured by kICS was significantly increased by elevation of cAMP with forskolin [31], whereas AQP2 showed a 10-fold reduction of the diffusion coefficient and that of AQP1 was unchanged (similar results in LLC-PK1 and MDCK cells, measured by FRAP) [21]. The diffusion coefficients of AQP2-EGFP and AQP1-EGFP measured by FRAP were down to fivefold lower than measured for AQP5-EGFP and AQP3-EGFP by kICS analysis. Differences could be expected as AQP2 and AQP1 measurements were carried out in the lateral membrane at cell-cell contacts of cells monolayers, whereas AQP5 and AQP3 diffusion was measured in subconfluent cells in either the free surface of the plasma membrane or by optical sectioning in the basal membrane [31]. Also, temperatures and acquisition rates differed as FRAP measurements were performed over 10 minutes with 25 ms integration at 1–2 s intervals at 23°C [21], whereas for kICS analysis imaging was performed in less than a minute at 37°C for 500 frames with 20 ms integration at 11.91 Hz (QDs) or at 30°C for 600 frames with 20 ms integration at 19.89 Hz (EGFP). In contrast, the diffusion coefficient of the plasma membrane targeting domain of the Lyn kinase, which localize to lipid rafts, diffused slowly (0.0093 ± 0.0088 μm^2^/s) and was unaltered by forskolin (0.0089 ± 0.0121 μm^2^/s) [31].

**Fig 3:**
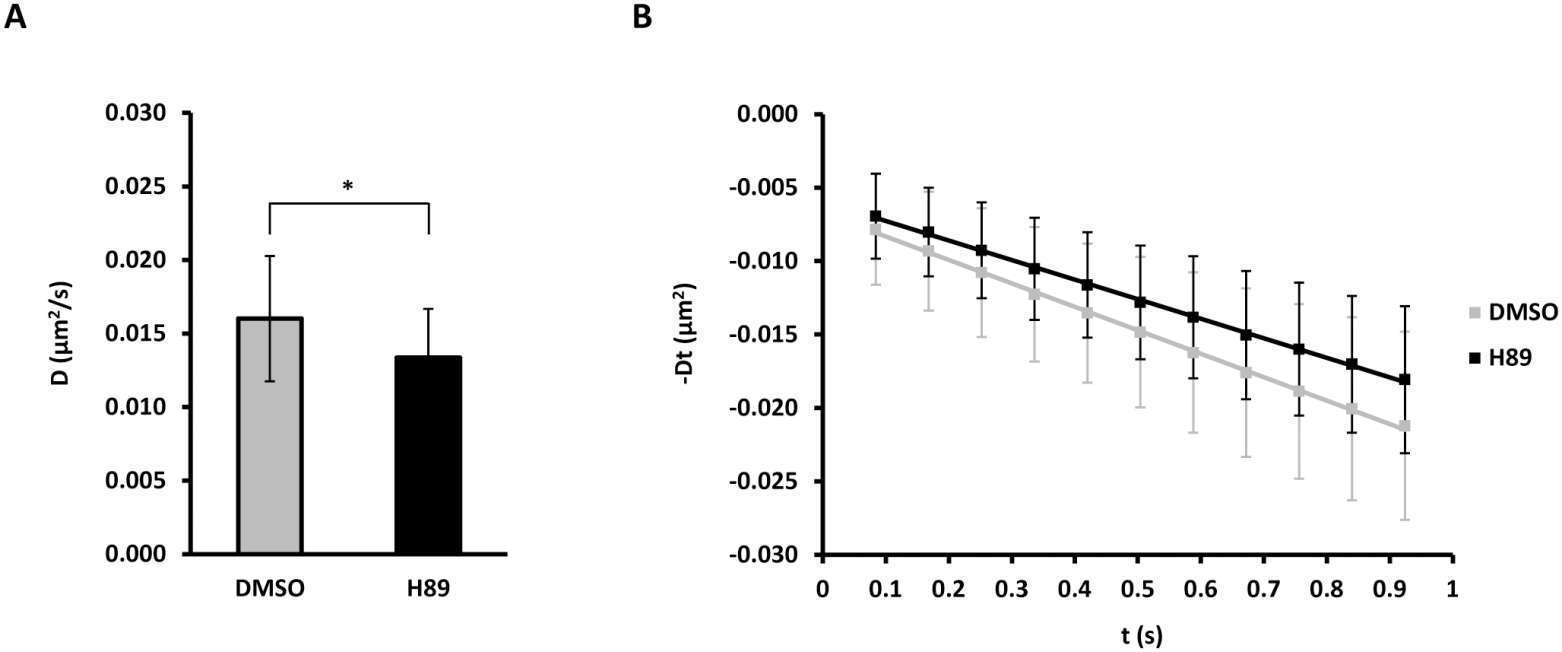
Inhibition of Protein Kinase A reduces the AQP5 diffusion coefficient. (A-B) MDCK cells stably expressing AQP5-myc-EGFP were treated with 30 μM H89 or vehicle (DMSO) for 30 min before labeling with QDs and followed by time-lapse microscopy with 20 ms integration for 500 frames at 11.91 Hz. Crops of image sequences were subjected to kICS analysis to determine average diffusion coefficients. (A) Graph showing the average diffusion coefficient, D, in μm^2^/s over all crops for cells treated with H89 and DMSO. (B) Diffusion plots showing time decay, -Dt, in μm^2^ versus time, t, in s averaged over all crops for H89 and DMSO treated cells. Values represent the mean ± standard deviation. * indicates *p* < 0.05.

To determine whether PKA activity also regulates AQP5 in the plasma membrane we measured the diffusion coefficient of AQP5 in the presence of the PKA inhibitor H89, which prevents long-term cAMP-induced increase of AQP5 expression levels [14, 15] and reported plasma membrane translocation [14, 15, 38]. After H89 treatment the diffusion coefficient of QD-labeled AQP5 was reduced to 83.5% of the control (0.0160 ± 0.0042 vs. 0.0134 ± 0.0033 μm^2^/s for DMSO and H89, respectively, *p* < 0.05) (Fig 3 and Table 1). Similar to forskolin, H89 had no apparent effect on AQP5 QD-labeling or subcellular localization (not shown), the latter in contrast to AQP2, where H89 induced intracellular localization despite forskolin stimulation [39].

Forskolin and H89 both reduce the diffusion coefficient of AQP5. It is possible that forskolin and H89 give rise to similar diffusion coefficients of AQP5 by acting through unrelated targets and mechanisms. Stimulation of cAMP and/or inhibition of PKA signaling with forskolin and H89, respectively, could regulate diffusion of AQP5 by both direct and indirect pathways. Besides being an inhibitor of PKA, H89 has many other cellular effects (reviewed in [40]), which could alternatively explain why we observe the same effect of forskolin and H89 on the AQP5 diffusion coefficient. Comparing with other inhibitors of PKA or using siRNA against PKA might rule out if the effect of H89 on AQP5 diffusion was not specifically due to PKA inhibition. However, that H89 blocks cAMP mediated effects on AQP5 membrane abundance and phosphorylation would seem to indicate that specific actions of PKA are inhibited [15, 17, 38]. To the best of our knowledge, there are no examples in the literature, which directly suggest that forskolin and H89, could not act oppositely on AQP5 in the plasma membrane, and yet result in the same average diffusion coefficient. FRAP measurements in CHO cells treated with forskolin, showed no significant change in the apparent diffusion coefficient of the human serotonin_1A_ (5-HT_1A_) receptor, even though the mobile fraction of the protein was significantly increased [41]. It is possible that mobile fractions and sub-diffusion modes of AQP5 differ between forskolin and H89 treated cells. This type of information is however, not given by kICS and would require other methods such as FRAP or SPT to determine.

### Phosphorylation at AQP5-T259 regulates the AQP5 diffusion coefficient

**Fig 4:**
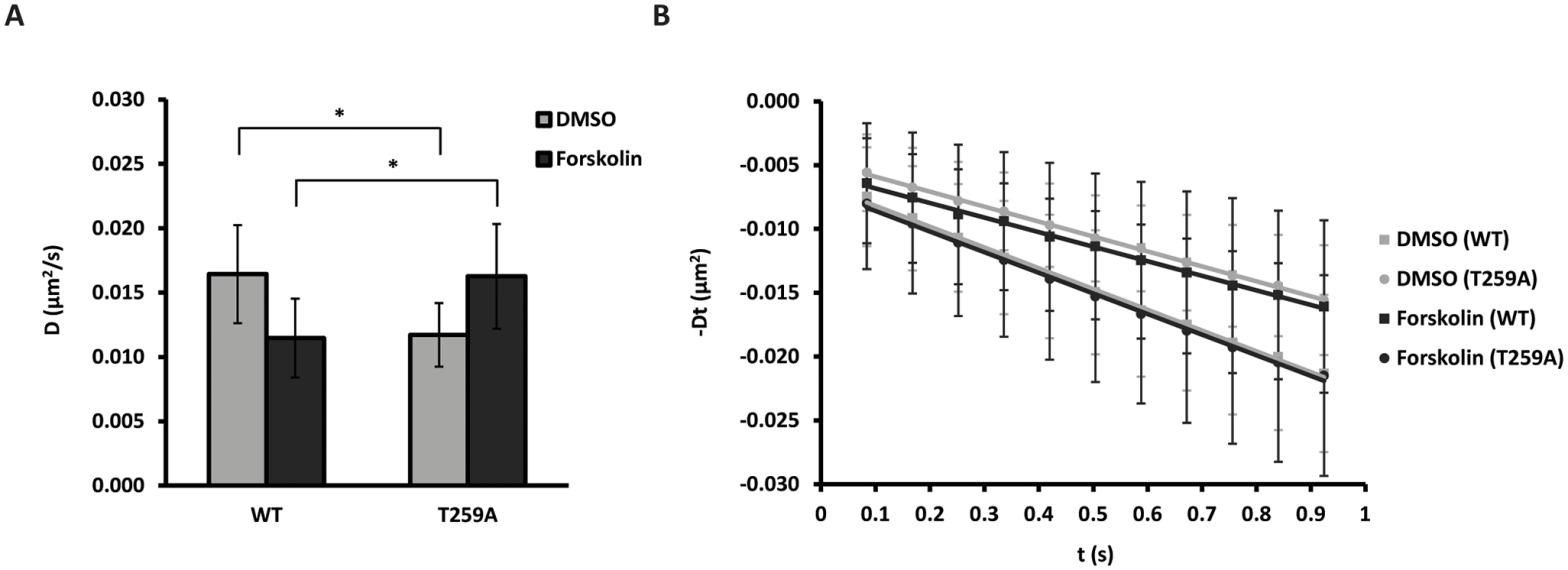
Phosphorylation of T259 regulates the AQP5 diffusion coefficient. (A-B) MDCK wt cells transiently transfected with AQP5-myc-EGFP wt and T259A were treated with 50 μM forskolin or vehicle (DMSO) for 30 min before labeling with QDs and followed by time-lapse microscopy with 20 ms integration for 500 frames at 11.91 Hz. Crops of image sequences were subjected to kICS analysis to determine average diffusion coefficients. (A) Graph showing the average diffusion coefficient, D, in μm^2^/s over all crops for cells treated with forskolin and DMSO. (B) Diffusion plots showing time decay, -Dt, in μm^2^ versus time, t, in s averaged over all crops for forskolin and DMSO treated cells. Values represent the mean ± standard deviation. * indicates *p* < 0.05.

PKA-dependent phosphorylation of AQP5-T259 has been identified in the plasma membrane, but does not appear to regulate AQP5 plasma membrane trafficking [17, 38]. The average diffusion coefficient of the QD-labeled phosphomutant mimicking constitutively non-phosphorylated AQP5 (AQP5-myc-T259A-EGFP) was 71.3% of the AQP5 wt control (0.0164 ± 0.0038 μm^2^/s vs. 0.0117 ± 0.0025 μm^2^/s for wt and T259A, respectively, *p* < 0.05) (Fig 4 and Table 1), whereas forskolin increased the diffusion coefficient to 141.7% of the AQP5 wt control (0.0115 ± 0.0031 μm^2^/s vs. 0.0163 ± 0.0041 μm^2^/s for wt and T259A, respectively, *p* < 0.05) (Fig 4 and Table 1). The T259A mutation had no apparent effect on AQP5 localization and extent of QD-labeling as compared to control (not shown).

These results show that AQP5-T259 phosphorylation alters diffusion. Shuttling of AQP5 in response to forskolin and H89 is not apparent in our cell culture system, suggesting that fundamental differences exist between regulation of AQP5 and AQP2. Membrane diffusion of an AQP2-S256A mutant was similar compared to AQP2 wt in control conditions, but was not slowed in response to cAMP [21]. However, it is further complicating that AQP2-S256 phosphorylation controls AQP2 plasma membrane association in a complex mechanism also involving a hierarchy of S261, S264, and S269 phosphorylation [18, 42, 43] as well as opposing K270 ubiquitination [44, 45]. Additional phosphorylation sites (including S231, S233, and T263; www.cbs.dtu.dk/services/NetPhos/) and ubiquitination sites (including K249, K257, and K258; www.ubpred.org) are predicted in the cytoplasmic tail of AQP5, but have not been validated experimentally. Our data thus support that AQP5-T259 phosphorylation does not mediate AQP5 trafficking [17, 38]. That cAMP mediated translocation of AQP5-T259A could be inhibited by H89 may indicate that another potential PKA-dependent phosphorylation site is involved [38]. Instead, AQP5-T259 phosphorylation may serve as part of a molecular switch involved in regulating diffusion of the protein within the plasma membrane.

### Calcium release and cholesterol depletion has no effect on the AQP5 diffusion coefficient

**Fig 5:**
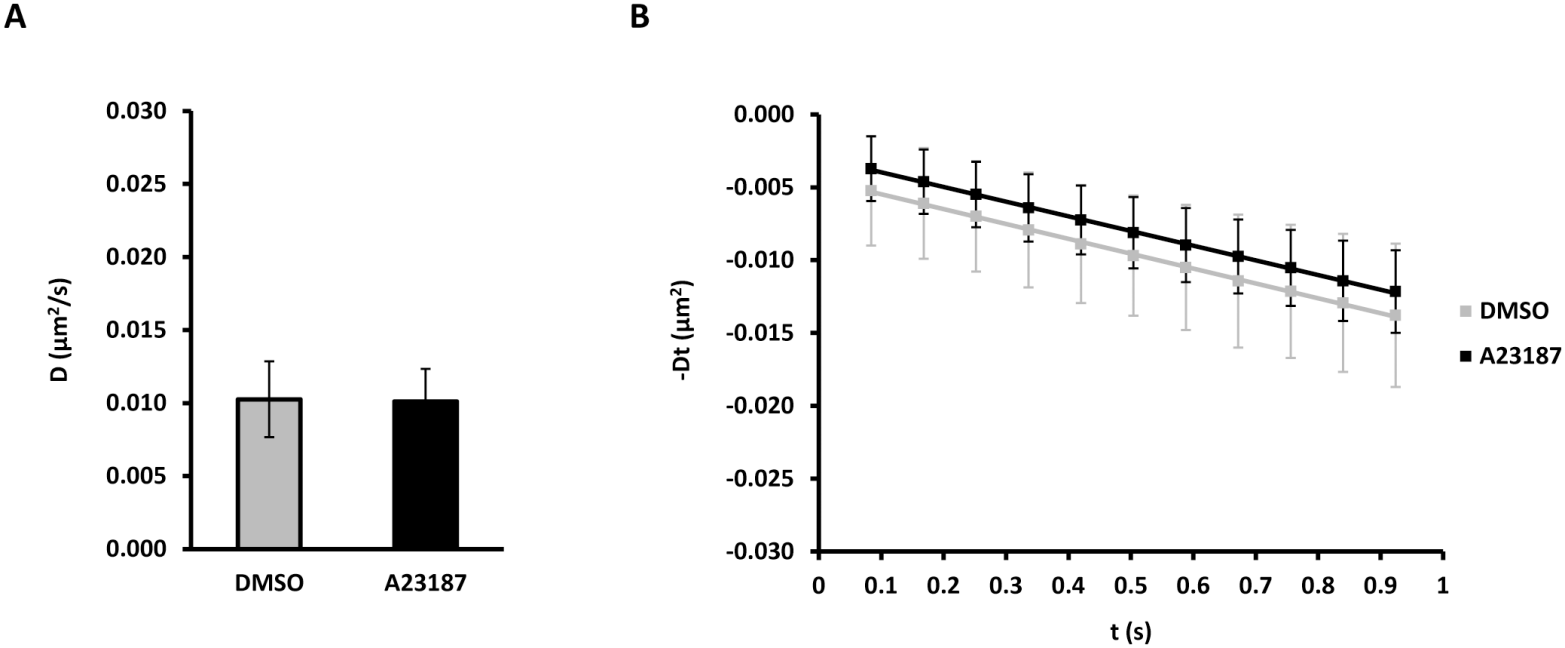
Intracellular calcium release does not change the AQP5 diffusion coefficient. (A-B) MDCK cells stably expressing AQP5-myc-EGFP, were labeled with QDs and either treated with 10 μM calcium ionophore A23187 or vehicle (DMSO) for 5 min, followed by time-lapse microscopy with 20 ms integration for 500 frames at 11.91 Hz. Crops of image sequences were subjected to kICS analysis to determine average diffusion coefficients. (A) Graph showing the average diffusion coefficient, D, in μm^2^/s over all crops for cells treated with A23187 and DMSO. (B) Diffusion plots showing time decay, -Dt, in μm^2^ versus time, t, in s averaged over all crops for A23187 and DMSO treated cells. Values represent the mean ± standard deviation.

Intracellular calcium release triggers glandular secretion and has been reported to cause transient translocation of AQP5 to the apical membrane in rat tissue slices and cell culture [46–50]. The calcium ionophore A23187 did not change the average diffusion coefficient of QD-labeled AQP5 (0.0103 ± 0.0026 μm^2^/s vs 0.0101 ± 0.0022 μm^2^/s for DMSO and A23187, respectively) (Fig 5 and Table 1). Similar to all previous treatments, calcium ionophore A23187 had no apparent effect on AQP5 subcellular localization (not shown).

**Fig 6:**
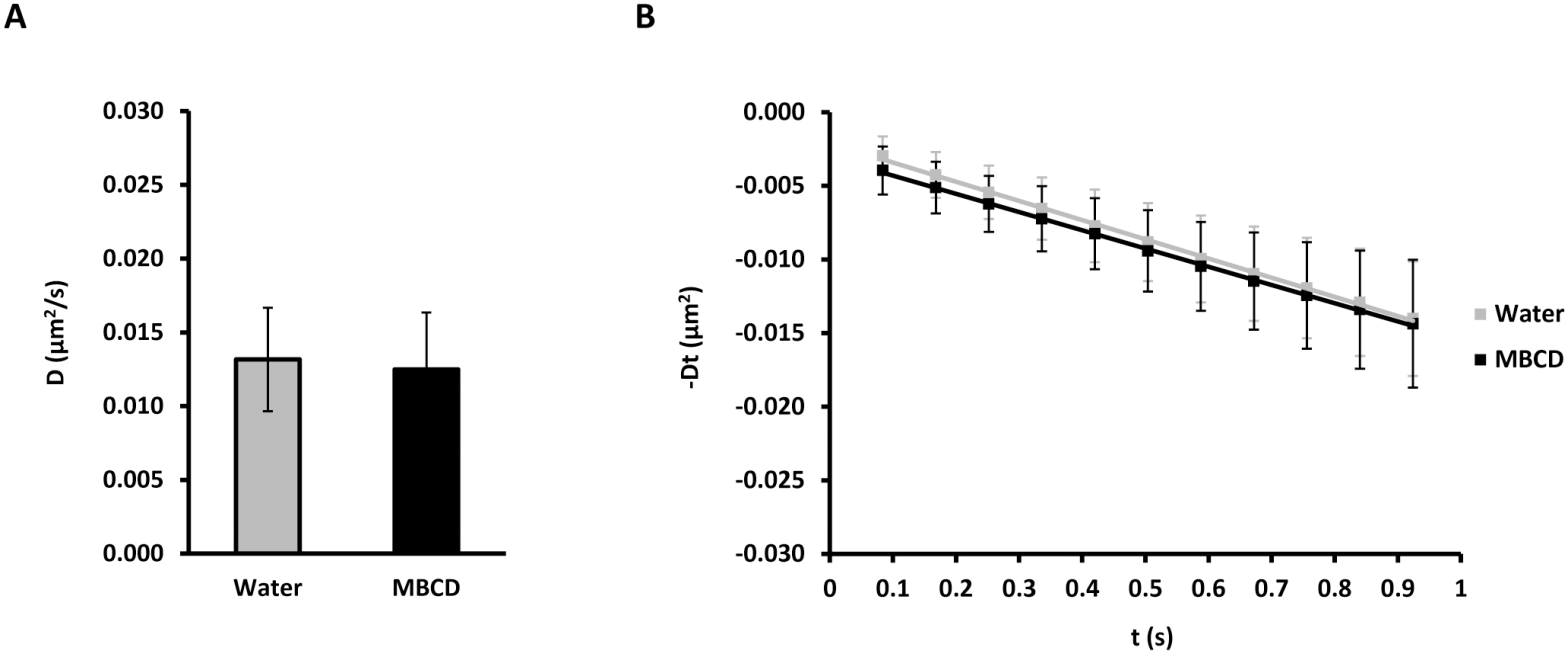
Depletion of cholesterol does not change the AQP5 diffusion coefficient. (A-B) MDCK cells stably expressing AQP5-myc-EGFP, were labeled with QDs and either treated with 5 mM MBCD or vehicle (water) for 15 min, followed by time-lapse microscopy with 20 ms integration for 500 frames at 11.91 Hz. Crops of image sequences were subjected to kICS analysis to determine average diffusion coefficients. (A) Graph showing the average diffusion coefficient, D, in μm^2^/s over all crops for cells treated with MBCD and water. (B) Diffusion plots showing time decay, -Dt, in μm^2^ versus time, t, in s averaged over all crops for MBCD and water treated cells. Values represent the mean ± standard deviation.

AQP5 has been detected in detergent resistant lipid raft membranes in rat parotid glands and moves into non-raft membrane domains upon translocation from intracellular sites to the apical plasma membrane after cholinergic stimulation and treatment with calcium ionophore A23187 [51]. The diffusion coefficient of QD-labeled AQP5 was unchanged by lipid raft disruption by cholesterol-depleting agent MBCD (0.0132 ± 0.0035 μm^2^/s vs 0.0125 ± 0.0039 μm^2^/s for water and MBCD, respectively) (Fig 6 and Table 1). In contrast, MBCD strongly reduced the diffusion of basolateral AQP3 in MDCK cells determined by kICS analysis [37] and the non-polarized AQP1 in COS-7 cells and MDCK cells when measured by QD single particle tracking [33], suggesting that maybe only a subset of AQPs are associated with lipid rafts in the plasma membrane and sensitive to cholesterol removal.

## Conclusions and Future Perspectives

We successfully measured the diffusion coefficients of AQP5 in MDCK epithelial plasma membranes by kICS analysis of time-lapse image sequences of QD- and EGFP-labeled AQP5. The average diffusion coefficient of AQP5 within the plasma membrane was regulated by cAMP signaling, PKA activity, and AQP5-T259 phosphorylation, but not transient calcium release and cholesterol depletion. This indicates for the first time that acute regulation of AQP5 takes place in the plasma membrane. Further investigations will be necessary to determine how cAMP and PKA dependent pathways and AQP5-T259 phosphorylation regulate diffusion and potentially modulate organization, protein and lipid interactions as well as turnover of AQP5 in epithelial plasma membranes. Single particle tracking approaches with faster acquisition rates and super-resolution microscopy may advance detection of AQP5 subpopulations and reveal the nanoscale organization of AQP5 in the plasma membrane.

## Supporting Information

S1 Fig DMSO dissolvent does not alter the AQP5 diffusion coefficient. (A-B) MDCK cells stably expressing AQP5-myc-EGFP were labeled with QDs and either left untreated or treated with DMSO (1:1000) for 30 min, followed by time-lapse microscopy with 20 ms integration for 500 frames at 11.91 Hz. Crops of image sequences were subjected to kICS analysis to determine average diffusion coefficients. (A) Graph showing the average diffusion coefficient, D, in μm^2^/s over all crops for untreated cells or cells treated with DMSO. (B) Diffusion plots showing time decay, -Dt, in μm^2^ versus time, t, in s averaged over all crops for untreated and DMSO treated cells. Values represent the mean ± standard deviation.
(TIF)

S2 Fig Increased cAMP reduces the AQP5 diffusion coefficient in non-labeled cells. (A-B) MDCK cells stably expressing AQP5-EGFP and AQP5-myc-EGFP were left untreated or treated with 50 μM forskolin or vehicle (DMSO) for 30 min and followed by time-lapse microscopy with 20 ms integration for 600 frames at 19.89 Hz. Crops of image sequences were subjected to kICS analysis to determine average diffusion coefficients. (A) Graph showing the average diffusion coefficient, D, in μm^2^/s over all crops for cells left untreated or treated with forskolin and DMSO. (B) Diffusion plots showing time decay, -Dt, in μm^2^ versus time, t, in s averaged over all crops for untreated cells and cells treated with forskolin and DMSO. Values represent the mean ± standard deviation. * indicates *p* < 0.05.
(TIF)
