# Supplementary figures and images for: Opposing Effects of cAMP and T259 Phosphorylation on Plasma Membrane Diffusion of the Water Channel Aquaporin-5 in Madin-Darby Canine Kidney Cells
Source: PLoS One. 2015 Jul 28;10(7):e0133324. doi: 10.1371/journal.pone.0133324 (PMC4517861; doi:10.1371/journal.pone.0133324)

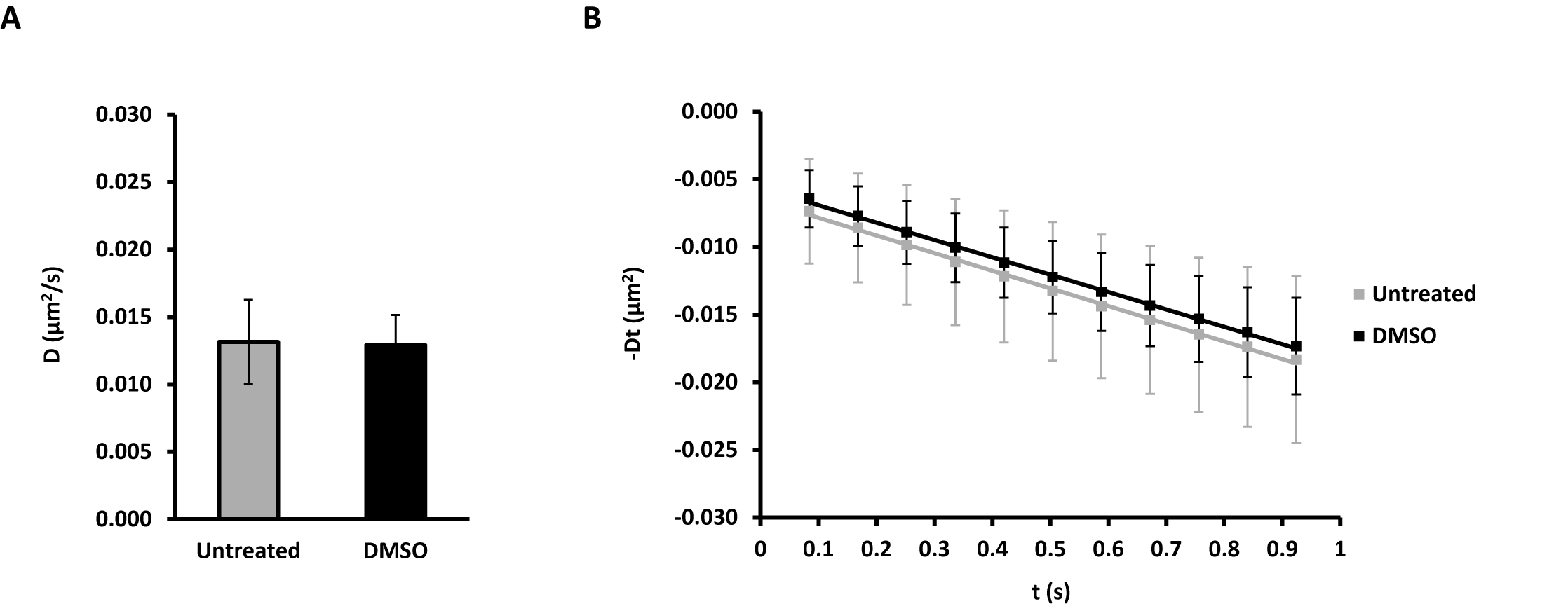

Supplement: S1 Fig — (A-B) MDCK cells stably expressing AQP5-myc-EGFP were labeled with QDs and either left untreated or treated with DMSO (1:1000) for 30 min, followed by time-lapse microscopy with 20 ms integration for 500 frames at 11.91 Hz. Crops of image sequences were subjected to kICS analysis to determine average diffusion coefficients. (A) Graph showing the average diffusion coefficient, D, in μm2/s over all crops for untreated cells or cells treated with DMSO. (B) Diffusion plots showing time decay, -Dt, in μm2 versus time, t, in s averaged over all crops for untreated and DMSO treated cells. Values represent the mean ± standard deviation. (TIF) [file pone.0133324.s001.tif]

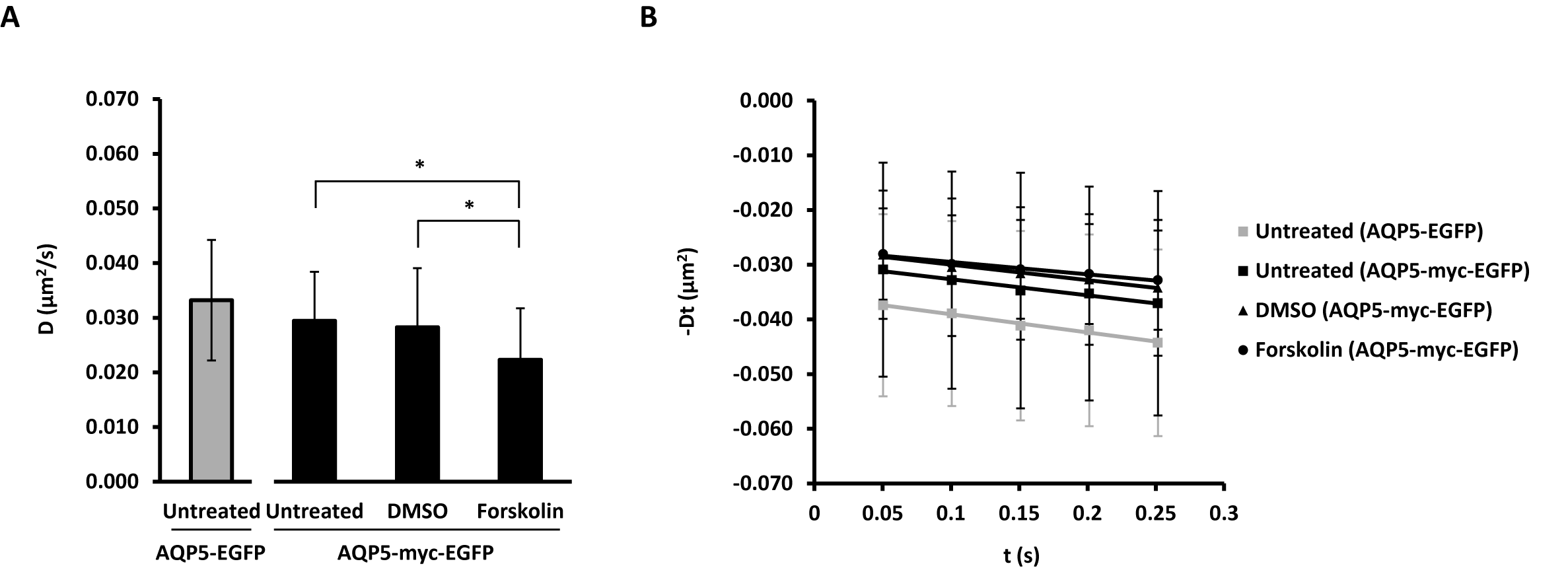

Supplement: S2 Fig — (A-B) MDCK cells stably expressing AQP5-EGFP and AQP5-myc-EGFP were left untreated or treated with 50 μM forskolin or vehicle (DMSO) for 30 min and followed by time-lapse microscopy with 20 ms integration for 600 frames at 19.89 Hz. Crops of image sequences were subjected to kICS analysis to determine average diffusion coefficients. (A) Graph showing the average diffusion coefficient, D, in μm2/s over all crops for cells left untreated or treated with forskolin and DMSO. (B) Diffusion plots showing time decay, -Dt, in μm2 versus time, t, in s averaged over all crops for untreated cells and cells treated with forskolin and DMSO. Values represent the mean ± standard deviation. * indicates p < 0.05. (TIF) [file pone.0133324.s002.tif]
